# Human colon organoid differentiation from induced pluripotent stem cells using an improved method

**DOI:** 10.1002/1873-3468.15082

**Published:** 2024-12-23

**Authors:** I‐Ting Lee, Yu Takahashi, Takashi Sasaki, Yoshio Yamauchi, Ryuichiro Sato

**Affiliations:** ^1^ Food Biochemistry Laboratory, Department of Applied Biological Chemistry, Graduate School of Agricultural and Life Sciences The University of Tokyo Japan; ^2^ Nutri‐Life Science Laboratory, Department of Applied Biological Chemistry, Graduate School of Agricultural and Life Sciences The University of Tokyo Japan

**Keywords:** BMP2 signaling, colon organoids, HOXD13, induced pluripotent stem cells, SATB2

## Abstract

The colonic epithelium plays a crucial role in gastrointestinal homeostasis, and colon organoids enable investigation into the molecular mechanisms underlying colonic physiology. However, the method for differentiating induced pluripotent stem cells (iPSCs) into human colon organoids (HCOs) is not necessarily standardized, and studies using HCOs are limited. This study refines the differentiation of HCOs by comparing two protocols reported in *Cell Stem Cell* and *Nature Medicine* journals. The former protocol, which uses transient bone morphogenetic protein 2 (BMP2) signaling activation, demonstrated superior efficacy in upregulating colon‐specific markers. Additionally, adenovirus‐mediated transduction of the transcription factors HOXD13 or SATB2 during hindgut endoderm development, together with BMP2 treatment, enhanced colonic identity, suggesting improved colonic maturation. This optimized protocol advances the generation of mature HCOs, offering a better model for investigating colonic epithelial biology and pathology.

## Graphical abstract

We compared established protocols to differentiate induced pluripotent stem cells into colon organoids and found that activation of bone morphogenetic protein 2 signaling induced gene expression of colon‐specific markers. Adenovirus‐mediated transduction of the transcription factors HOXD13 or SATB2 further enhanced colonic gene expression, suggesting improved colonic maturation. This new differentiation method may contribute to the investigation of colonic epithelial biology and pathology.

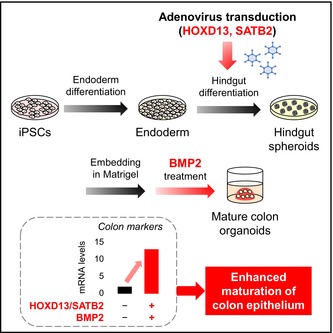

## Abbreviations

**Ad**, adenovirus

**AP**, anterior–posterior

**BMP**, bone morphogenetic protein

**CSC**, cell stem cell

**CsCl**, cesium chloride

**FBS**, fetal bovine serum

**FGF**, fibroblast growth factor

**GFP**, green fluorescent protein

**HCO**, human colonic organoid

**HOX**, homeobox

**HSIO**, human small intestinal organoid

**iPSC**, induced pluripotent stem cell

**MOI**, multiplicity of infection

**NM**, nature medicine

**qRT‐PCR**, quantitative reverse transcription‐polymerase chain reaction

**SEM**, standard error of the mean

**TCID50**, median tissue culture infectious dose

The colonic epithelium is a complex biochemical interface that orchestrates diverse physiological processes crucial for gastrointestinal homeostasis. In contrast to the primary role of the small intestine in nutrient absorption, the colon reabsorbs water and electrolytes, stores fecal matter, synthesizes vitamin K, and hosts a diverse microbiome that mediates complex interactions between gut microbes and host immunity [1, 2]. The increasing prevalence of colon‐related pathologies, including colorectal cancer, colitis, and irritable bowel syndrome, underscores the need to elucidate the molecular mechanisms underlying the colonic physiology. However, research on colon biology and pathology is limited by available models. Traditional approaches relying on animal studies or cancer‐derived cell lines often fail to accurately replicate human intestinal physiology owing to interspecies differences and abnormal characteristics of transformed cells [3]. Colonic organoids have emerged as a promising model system, offering a physiologically relevant context for investigating the complex biochemistry of the colon epithelium. These three‐dimensional cultures demonstrate self‐organization, self‐renewal, and differentiation capabilities, closely mimicking the molecular complexity of their *in vivo* counterparts [4, 5].

At the molecular level, colonic epithelial development is governed by complex signaling cascades. During embryogenesis, the colonic epithelium differentiates from the definitive endoderm and undergoes patterning in the hindgut along the anterior–posterior (AP) axis [6, 7]. This process is orchestrated by intricate interactions between the endoderm and the underlying mesoderm *via* several key molecular pathways, notably WNT, bone morphogenetic protein (BMP)/SMAD, Notch, and Hedgehog signaling systems [8, 9]. These pathways regulate the gene expression profiles and cellular behaviors that ultimately define colonic epithelial identity.

Recent advances in stem cell biology have enabled *in vitro* recapitulation of developmental events using induced pluripotent stem cells (iPSCs). Two established protocols have emerged for guiding hindgut spheroids toward the colonic fate, each employing distinct molecular approaches. The *Cell Stem Cell* (CSC) protocol utilizes transient activation of BMP2 signaling in the hindgut endoderm [10], whereas the *Nature Medicine* (NM) protocol relies on sustained WNT activation coupled with BMP inhibition [11]. Despite their divergent approaches to manipulating these molecular pathways, both protocols have been successful in generating colonic organoids. However, a comprehensive molecular characterization and comparison of the maturity of the resulting organoids remain to be conducted.

HOXD13 and SATB2 are two essential transcription factors that orchestrate complex molecular events crucial for intestinal specification and homeostasis. HOXD13 belongs to the homeobox (HOX) gene family and is an evolutionarily conserved regulator responsible for patterning regional specifications during embryogenesis [12]. HOX genes are organized in clusters on chromosomes, with their 3′ to 5′ order corresponding to their colinear expression along the AP axis, guiding proper organ formation from head to tail [13, 14]. While nearly all HOX genes are expressed in the adult colonic epithelium, the posterior part of the gastrointestinal tract [14, 15], mounting evidence points to the importance of HOXD13 not only during intestinal specification in embryonic hindgut development, but also in adulthood. Misexpression of Hoxd13 in the midgut mesoderm prompts the endoderm to adopt hindgut‐like features through epithelial‐mesenchymal interactions [16]. A loss‐of‐function mutation of Hoxd13 in mouse embryos results in defective muscular and epithelial layers of the rectum [17]. Intriguingly, while other posterior HOX genes show dynamic spatiotemporal expression, HOXD13 maintains consistent activation from early embryonic hindgut development through the adult rectal region [18]. SATB2, a colon‐restricted transcription factor, is a key regulator of colonic epithelial identity and orchestrates both the initial formation and long‐term maintenance of colonic epithelium. During embryogenesis, SATB2 marks the posterior endoderm, establishing a clear boundary with the GATA4‐positive anterior intestine, which is a division that persists into adulthood [10]. Beyond embryonic patterning, SATB2 actively maintains colonic identity by suppressing small intestine‐specific gene programs and by preserving colonic stem cell characteristics. The critical nature of this regulation is evident when SATB2 is lost: the colonic epithelium undergoes a remarkable fate shift, adopting ileal characteristics through extensive enhancer remodeling [19]. Recent studies have further implicated SATB2 dysregulation in colitis and colorectal cancer pathogenesis, hinting at its broader clinical relevance [20, 21, 22]. Collectively, these findings imply that HOXD13 and SATB2 are crucial regulators of proper development, maintenance, and homeostasis of the colonic epithelium throughout life.

In this study, we aimed to refine the molecular approach for differentiating mature iPSC‐derived human colonic organoids (HCOs). We first conducted a comparative analysis of the two established protocols to evaluate their efficacy in recapitulating the molecular signatures of the colonic epithelium. Subsequently, we investigated the effects of the transient expression of colon‐specific genes, HOXD13 and SATB2, during the differentiation process. By manipulating these key molecular regulators, we sought to enhance colonic identity and promote organoid maturation, thereby developing a sophisticated *in vitro* model for studying colonic epithelial biochemistry and physiology.

## Materials and methods

### Cell culture

The human embryonic kidney cell line HEK293A (RRID: CVCL_6910) and hepatocellular carcinoma cell line HepG2 (RRID: CVCL_0027), which were obtained from Thermo Fisher Scientific (Waltham, MA, USA) and the American Type Culture Collection (Manassas, VA, USA), respectively, were maintained in high‐glucose DMEM supplemented with 10% (v/v) fetal bovine serum (FBS), 100 units·mL^−1^ penicillin, and 100 units·mL^−1^ streptomycin.

The human iPS cell line TkDN4‐M (RRID: CVCL_RJ58) was supplied by the Stem Cell Bank (The University of Tokyo) and cultured as colonies in complete Essential 8 medium (Thermo Fisher Scientific) on plates pre‐coated with vitronectin (Thermo Fisher Scientific). TkDN4‐M was passaged at a 1 : 2 to 1 : 5 ratio using 0.5 mm EDTA at 85% confluence. Y‐27632 (10 μm) was added for the first 24 h after passage. The medium was refreshed every day.

No mycoplasma contamination was detected using the MycoAlert™ Mycoplasma Detection Kit (Lonza, Basel, Switzerland). All cell lines were authenticated by their morphology, growth efficiency, unique capacity (adenovirus production, insulin responsiveness, and differentiation), and specific gene expression profiles.

All the cell cultures were maintained at 37 °C in a humidified atmosphere containing 5% CO_2_.

### Differentiation of iPSC‐derived organoids

TkDN4‐M cells were seeded on Matrigel hESC‐qualified matrix (Corning, Corning, NY, USA)‐coated plates 1 day prior to differentiation. For HCO generation, TkDN4‐M cells were progressively differentiated into definitive endoderm and hindgut spheroids following established protocols [10, 11]. On day 8 of differentiation, spheroids were collected and embedded in a Matrigel matrix (Corning) in the center of the multidish wells. Spheroids were cultured in the colon development medium until maturation (day 28 in the CSC protocol and day 48 in the NM protocol). The medium was refreshed every 3–4 days. Upon reaching confluence, organoids were resuspended in Matrigel droplets at a 1 : 5 to 1 : 10 passage ratio. For human small intestine organoid (HSIO) generation, TkDN4‐M cells were differentiated following a previously established protocol [23]. The composition of the differentiation medium used in each step is summarized in Table S1. For organoid harvest, differentiated organoids were released from Matrigel using Cell Recovery Solution (Corning) by incubation on ice for 30 min. After strong pipetting, the organoid suspension was transferred to a 15‐mL centrifuge tube and kept on ice for an additional 10 min to allow organoids to settle, while non‐organoid cells remained in suspension. After removal of the supernatant, the pelleted organoids were processed for RNA extraction or immunofluorescence staining.

### Generation of recombinant adenoviruses

Recombinant adenoviruses were generated using the ViraPower Adenoviral Expression System (Thermo Fisher Scientific), according to the manufacturer's instructions. Briefly, HOXD13 and SATB2 genes were tagged with a FLAG sequence at the C‐terminal site, a modification previously shown not to affect their functions [24, 25, 26], cloned into the entry vector pENTR1A (Thermo Fisher Scientific), and transferred into the adenoviral vector pAd/CMV/V5‐DEST (Thermo Fisher Scientific) using the Gateway™ LR Clonase™ II Enzyme mix (Thermo Fisher Scientific). Purified plasmid DNA was transfected into HEK293A cells using Lipofectamine 3000 (Thermo Fisher Scientific) to produce crude viral stocks. These stocks were diluted 1 : 100 and used to transfect HEK293A cells 2–3 times to generate higher‐titer adenoviral stocks. The adenoviral stocks were purified and concentrated by cesium chloride (CsCl) density gradient ultracentrifugation as previously described [27].

### Titer determination

Adenoviral stock titers were determined using the median tissue culture infectious dose (TCID50) assay. A 96‐well collagen‐coated plate (AGC Techno Grass, Shizuoka, Japan) was prepared with 50 μL DMEM supplemented with 5% FBS per well. The viral stock was serially diluted with DMEM supplemented with 5% FBS (1 × 10^−4^ to 1 × 10^−9^). The initial dilution (25 μL) was then added to the wells of the first column of the plate. A sequential 1 : 3 dilution of the viral stock was performed by transferring 25 μL between the adjacent columns from 1st to 11th. The 12th column received no viral solution and served as a negative control. HEK293A cells at 85% confluence in a 100‐mm dish were prepared for each adenovirus titer test. The cells were trypsinized and seeded (50 μL per well) in each well of a 96‐well plate. On days 4 and 7 post‐infection, 50 μL complete medium (DMEM supplemented with 10% FBS) was added to each well. On day 12, cytopathic effect was assessed using the CellTiter‐Fluor Cell Viability Assay (Promega, Madison, WI, USA). Fluorescence was measured using a Tristar Multiple Reader (Berthold Technologies, Bad Wildbad, Baden‐Württemberg, Germany), and the titer was calculated statistically using Käber's formula:

$$\text{TCID}50=\begin{array}{l} \text{(dilution rate in the first column)}\\ \times {\text{(dilution rate)}}^{\Sigma -0.5} \end{array}$$

where Σ is the total sum of (number of wells showing cytopathic effects)/(number of wells) at each dilution step.

### Adenoviral transduction of HepG2 cells

HepG2 cells were seeded in 12‐well plates at a density of 1 × 10^5^ cells per well 1 day before transduction. Adenovirus was introduced to the culture medium at the indicated multiplicity of infection (MOI). After 24 h, the medium was replaced with a normal growth medium. Cells were harvested 48 h post‐transduction for Immunoblot or quantitative reverse transcription‐polymerase chain reaction (qRT‐PCR) analysis.

### Adenoviral transduction of hindgut endoderm

For adenoviral transduction during hindgut differentiation, TkDN4‐M cells were subjected to HCO differentiation. On day 5, one well of the cells was trypsinized for cell counting. The required volume of viral suspension, calculated based on the determined MOI, was added to day 5 hindgut differentiation medium. The medium was replaced after 24 h. The control was defined as the no‐infection group in which an equivalent volume of base medium was added. On day 8 of differentiation, spheroids were harvested, and the underlying cells were collected to confirm transduction efficiency.

### RNA extraction and qRT‐PCR

Total cellular RNA was extracted using ISOGEN (Nippon Gene, Toyama, Japan). Reverse transcription was performed using a High‐Capacity cDNA Reverse Transcription Kit (Thermo Fisher Scientific). mRNA levels were quantified using a StepOnePlus real‐time PCR system (Thermo Scientific) with Fast Start Universal SYBR Green Master Mix (Roche, Basel, Switzerland) or TaqMan Gene Expression Master Mix (Thermo Fisher Scientific). All mRNA levels were normalized to 18s ribosomal RNA levels. Primers and probes (Integrated DNA Technologies, Coralville, IA, USA) used in this study are listed in Table S2.

### Immunoblot analysis

Cell lysates were extracted with RIPA buffer (50 mm Tris/HCl, pH 7.4, 1 mm EDTA, 150 mm NaCl, 1% Nonidet P‐40, 0.25% deoxycholate) supplemented with 1% protease inhibitor cocktail (Nacalai Tesque, Kyoto, Japan). The protein concentration was quantified using the Pierce BCA Protein Assay (Thermo Fisher Scientific). The samples were subjected to SDS/polyacrylamide gel electrophoresis and transferred onto polyvinylidene difluoride membranes (Merck Millipore, Billerica, MA, USA). Membranes were blocked in 5% (w/v) skim milk in Tris‐buffered saline with Tween 20 (20 mm Tris/HCl, pH 7.6, 137 mm NaCl, 0.1% Tween 20) for 1 h at room temperature, then probed with primary antibodies against green fluorescent protein (GFP) (Abcam, Cambridge, UK, ab6556, 1 : 1000), FLAG (Sigma‐Aldrich, St. Louis, MO, USA, F7425, 1 : 1000), and β‐Actin (Sigma‐Aldrich, A5441, 1 : 1000) overnight at 4 °C, followed by secondary antibodies for 1 h at room temperature. Signals were detected using Amersham ECL Western Blotting Detection Reagent (Cytiva, Tokyo, Japan) and visualized using a FUSION SOLO S chemiluminescence imaging system (Vilber Lourmat, Collégien, France). β‐actin served as an internal control.

### Immunofluorescence staining

Differentiated organoids were fixed with fixation buffer (BD Biosciences, San Jose, CA, USA) for 30 min at room temperature and washed with BD Perm/Wash buffer (BD Biosciences). The organoids were then incubated overnight at 4 °C with primary antibodies against Ki‐67 (BD Pharmingen, San Diego, CA, USA, 550609, 1 : 50), MUC2 (Cell Signaling Technology, Danvers, MA, USA, sc‐7314, 1 : 50), LYZ (Dako, Glostrup, Denmark, A0099, 1 : 10), and GATA4 (Cell Signaling Technology, sc‐25310, 1 : 100). Following primary antibody incubation, the samples were treated with secondary antibodies (goat anti‐mouse Alexa Fluor 488 or goat anti‐rabbit Alexa Fluor 568, Thermo Fisher Scientific, 1 : 500) for 3 h at 4 °C. DAPI staining was performed for 10 min at room temperature. Between each antibody treatment, organoids were washed three times with BD Perm/Wash buffer. Stained organoids were mounted using Fluoromount (Diagnostic BioSystems, Pleasanton, CA, USA) and imaged under an all‐in‐one fluorescence microscope equipped with structured illumination (Keyence, Osaka, Japan).

### Statistical analysis

Data are representative of at least two independent experiments with biological triplicates and are presented as mean ± standard error of the mean (SEM). Statistical analyses were conducted using the Tukey's test, as indicated. Differences were considered statistically significant at *P* < 0.05.

## Results

### Differential outcomes of CSC and NM protocols in HCO differentiation

**Fig. 1:**
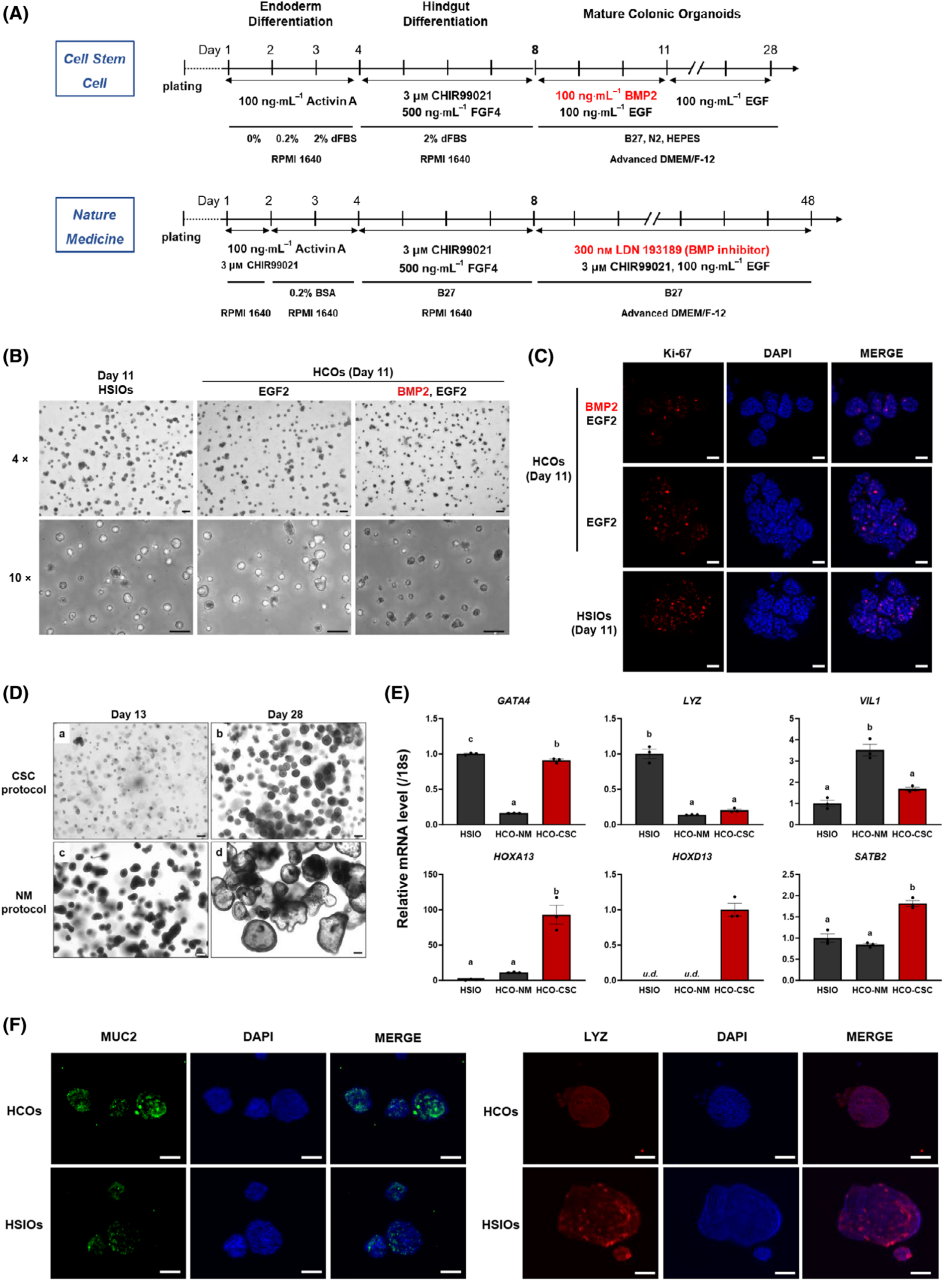
Comparative analysis of CSC and NM protocols for HCO differentiation from human induced pluripotent stem cells. (A) Schematic diagram of differentiation of TkDN4‐M into HCOs using the CSC and NM protocols. (B, C) Effects of BMP2 on cell proliferation. Bright‐field images (B) and immunofluorescence staining of Ki‐67 (C) in spheroids on day 11 derived from HSIOs or HCOs, with or without 3‐day BMP2 treatment. Scale bars: 200 μm (B) and 20 μm (C). (D) Bright‐field images of organoids on days 13 and 28 from CSC (a, b) and on days 13 and 28 from NM protocol (c, d). Representative images from NM protocol on day 28 are shown to enable morphological comparison with CSC‐HCOs, though NM‐HCOs continue maturation until day 48. Scale bar: 200 μm. (E) RT‐qPCR analysis of small intestine (*GATA4* and *LYZ*), intestinal epithelium (*VIL1*), and colon (*HOXA13*, *HOXD13*, and *SATB2*) markers in organoids (day 28 CSC‐HCOs, day 48 NM‐HCOs, and HSIOs). Experiments were performed independently three times (*n* = 3) with three biological replicates. Data normalized to 18s rRNA are shown as the mean ± SEM. Statistical analysis was performed using the Tukey's test (distinct letters, *P* < 0.05). ‘*u.d*.’ indicates ‘undetectable’. The closed black circles in the graph indicate individual data points. (F) Immunofluorescence staining of LYZ and MUC2 in HSIOs or HCOs derived from the CSC protocol. Scale bar: 20 μm.

The human iPSC line TkDN4‐M was subjected to differentiation protocols derived from CSC [10] and NM [11] journals to generate HCOs, with iPSC‐derived HSIOs serving as controls (Fig. 1A). During differentiation, we observed that the three‐day BMP2 treatment specified in the CSC protocol resulted in a marked reduction in spheroid size (Fig. 1B) and decreased Ki‐67^+^ proliferative cell populations within the spheroids (Fig. 1C) compared to both HSIOs or HCOs that did not receive BMP2 treatment. This finding aligns with the role of BMP signaling in inhibiting cell proliferation [28, 29]. Consistent with this result, on day 28, upon completion of the CSC protocol‐directed differentiation, CSC‐HCOs exhibited a much smaller size compared to NM‐HCOs (Fig. 1D). Notably, the size of NM‐HCOs was comparable between day 28 and day 48, when differentiation was completed using the NM protocol. To characterize differentiation outcomes, RT‐qPCR analysis was performed to examine the expression of small intestine‐specific (*GATA4* and *LYZ*), colon‐specific (*HOXA13*, *HOXD13*, and *SATB2*), and general intestinal epithelial (*VIL1*) marker genes. While CSC‐HCOs exhibited a more modest reduction in GATA4 expression relative to HSIOs compared to NM‐HCOs, they demonstrated markedly enhanced expression of colonic markers (Fig. 1E). These findings suggest that the CSC protocol yields HCOs with pronounced colonic epithelial characteristics, indicating that BMP2 signaling is a key determinant of posterior fate specification during HCO differentiation. Given previous reports that BMP2 signaling activation in the CSC protocol promotes mesenchymal commitment [10], we investigated the spatial distribution of GATA4 expression through immunofluorescence analysis, which revealed comparable GATA4‐positive staining patterns within the epithelial compartments of both HSIOs and HCOs (Fig. S1). Further immunofluorescence studies demonstrated elevated levels of the colonic goblet cell marker MUC2, accompanied by reduced LYZ protein expression in CSC‐HCOs compared with HSIOs (Fig. 1F).

### Optimization of HCO differentiation protocol

To enhance HCO differentiation efficiency, we devised a differentiation protocol to differentiate iPSCs into definitive endoderm. We previously reported [23] that supplementation with Wnt3a and fibroblast growth factor (FGF) 2 during the initial differentiation phase (days 1–3) enhanced the efficiency of hindgut spheroid formation (Fig. S2A). This modification resulted in augmented spheroid production without significantly affecting the expression of the marker genes (Fig. S2B, D). Having previously used Wnt3a instead of CHIR99021 for HSIO generation during hindgut differentiation (days 4–7), we tested this substitution in the CSC protocol for HCO generation (Fig. S2A). The results indicated that spheroids generated with Wnt3a during this phase were significantly smaller and exhibited poor survival during the subsequent 3‐day BMP2 treatment, which is essential for colonic fate specification (Fig. S2B,C). The reduced spheroid size, compounded by BMP2‐induced cell death, led to a drastic reduction in the number of surviving organoids, rendering this approach unsuitable for efficient HCO generation. Based on these findings, supplementation with Wnt3a and FGF2 during the initial differentiation phase (days 1–3) and CHIR99021 during the hindgut differentiation phase (days 4–7) was adopted for all subsequent differentiation experiments.

**Fig. 2:**
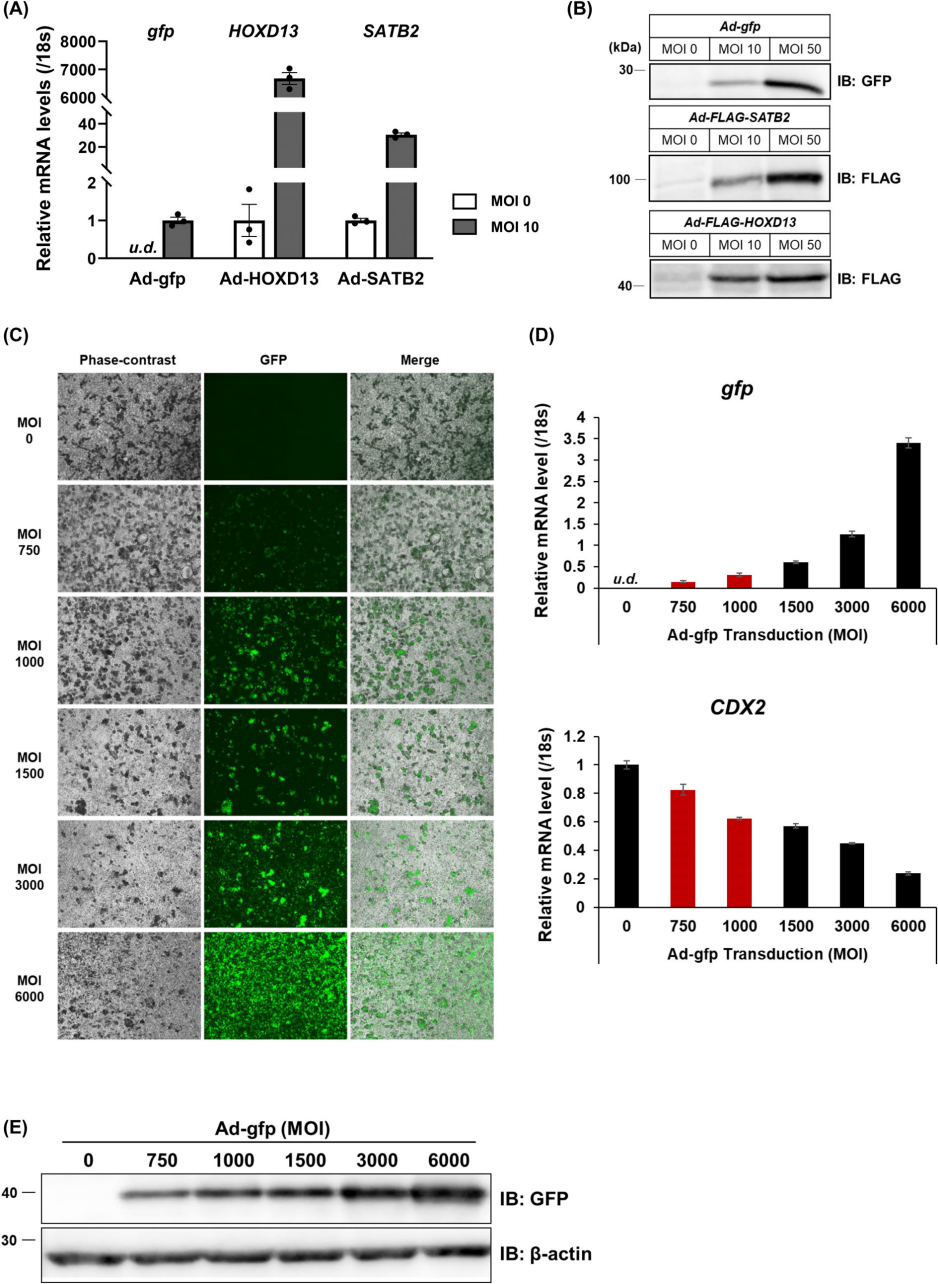
Optimization of adenovirus (Ad)‐mediated exogenous gene expression for HCO differentiation. (A, B) HepG2 cells transduced with Ad‐gfp, Ad‐HOXD13, or Ad‐SATB2 at a MOI of 10 or 50 for 24 h. Transgene expression assessed by (A) RT‐qPCR and (B) immunoblotting 48 h after transduction. (A) Data normalized to 18s rRNA are shown as the mean ± SEM. The closed black circles in the graph indicate individual data points. (C–E) TkDN4‐M‐derived differentiated cells transduced with Ad‐gfp (MOI of 750–6000) on day 5 for 24 h. Analyses were performed 48 h post‐transduction: (C) GFP fluorescence and phase‐contrast images. Scale bar: 200 μm. (D) RT‐qPCR analysis of *gfp* and *CDX2* expression. Experiments were performed independently three times (*n* = 3) with three biological replicates. Data normalized to 18 s rRNA are shown as the mean ± SEM. (E) Immunoblotting analysis of GFP expression. ‘*u.d*.’ indicates ‘undetectable’.

Recombinant adenoviruses expressing colon‐specific transcription factors HOXD13 and SATB2, which are pivotal regulators of posterior patterning during embryogenesis [6], were generated. These constructs, which incorporated FLAG tags, were purified *via* cesium chloride density gradient ultracentrifugation to achieve high titers (Fig. S3A). The efficacy of viral transduction was verified by RT‐qPCR and immunoblot analyses in HepG2 cells, confirming the elevated expression of each exogenous gene at both the mRNA and protein levels (Fig. 2A,B).

We established optimal adenoviral transduction at day five of differentiation, coinciding with peak CXADR (adenovirus receptor) expression in progenitor cells and initial CDX2 expression (Fig. S3B). The adenoviral system generally provides transient expression peaking at 48–72 h post‐transduction, strategically aligning with the critical window of hindgut specification (days 5–8) to promote efficient differentiation toward the colonic epithelium.

Adenovirus (Ad)‐GFP was used at MOIs ranging from 750 to 6000 to determine the optimal MOI. Dose‐dependent increases in GFP fluorescence, mRNA levels, and protein expression were observed, concomitant with a reduction in CDX2 expression, suggesting hindered differentiation efficiency of the hindgut endoderm (Fig. 2C–E). Maximal transduction efficiency was achieved at an MOI of 1000, and severe cytotoxicity was observed at an MOI of 3000. Consequently, MOIs of 750 and 1000 were selected for subsequent experiments.

### Impact of HOXD13 and SATB2 transduction on HCO specification

We investigated whether the refined modification of the CSC protocol, with supplementation of Wnt3a and FGF2 during the initial differentiation phase (days 1–3) and transduction with Ad‐HOXD13 or Ad‐SATB2 at MOIs of 750 and 1000 on day 5, respectively, improved the maturity and efficiency of HCO differentiation.

**Fig. 3:**
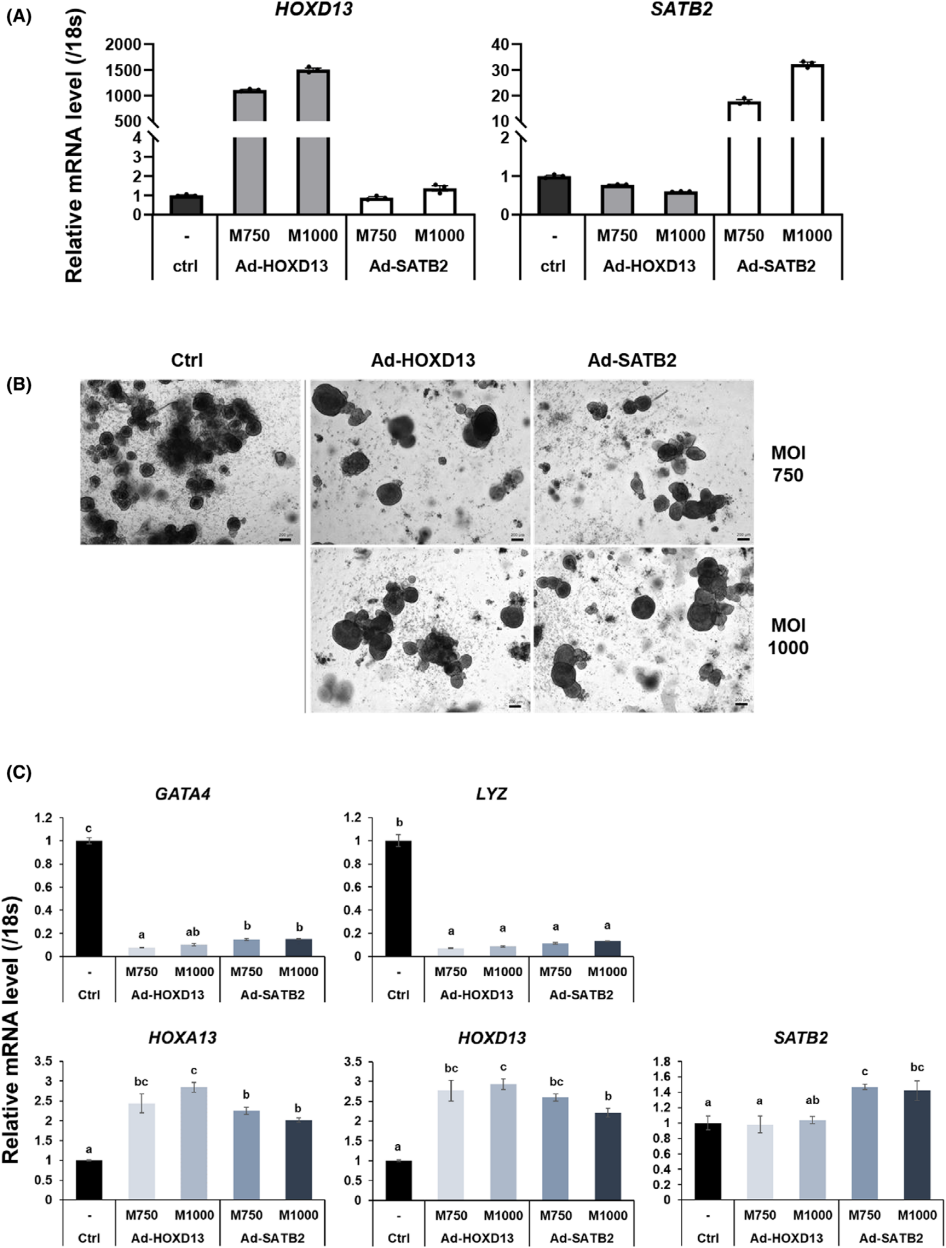
Adenovirus (Ad)‐mediated HOXD13 and SATB2 expression enhances colonic identity in developing HCOs. (A) RT‐qPCR analysis of transgene expression in the developing hindgut endoderm after Ad‐HOXD13 and Ad‐SATB2 transduction (MOI of 750 and 1000) on day 5. The samples were harvested on day 8. Experiments were performed independently twice (*n* = 2) with three biological replicates. Data normalized to 18s rRNA are shown as the mean ± SEM. The closed black circles in the graph indicate individual data points. (B) Bright‐field images of Day 28 mature HCOs. Scale bar: 200 μm. (C) RT‐qPCR analysis of small intestine (*GATA4* and *LYZ*) and colon (*HOXA13*, *HOXD13*, and *SATB2*) markers on day 28 HCOs, which were differentiated using the CSC protocol with Wnt3a and FGF2 supplementation during the initial endoderm patterning stage. Statistical analysis was performed using the Tukey's test (distinct letters, *P* < 0.05).

The elevated expression of the transgenes was confirmed (Fig. 3A) and transduced spheroids successfully developed into mature HCOs without apparent morphological alterations (Fig. 3B). RT‐qPCR analysis revealed that adenoviral transduction of either HOXD13 or SATB2 resulted in the downregulation of small intestine markers (*GATA4* and *LYZ*) and upregulation of colon markers (*HOXA13*, *HOXD13*, and *SATB2*), indicating enhanced colonic identity in transduced organoids (Fig. 3C).

### Promotion of colonic fate by the combination of HOXD13 or SATB2 transduction with BMP2 treatment

**Fig. 4:**
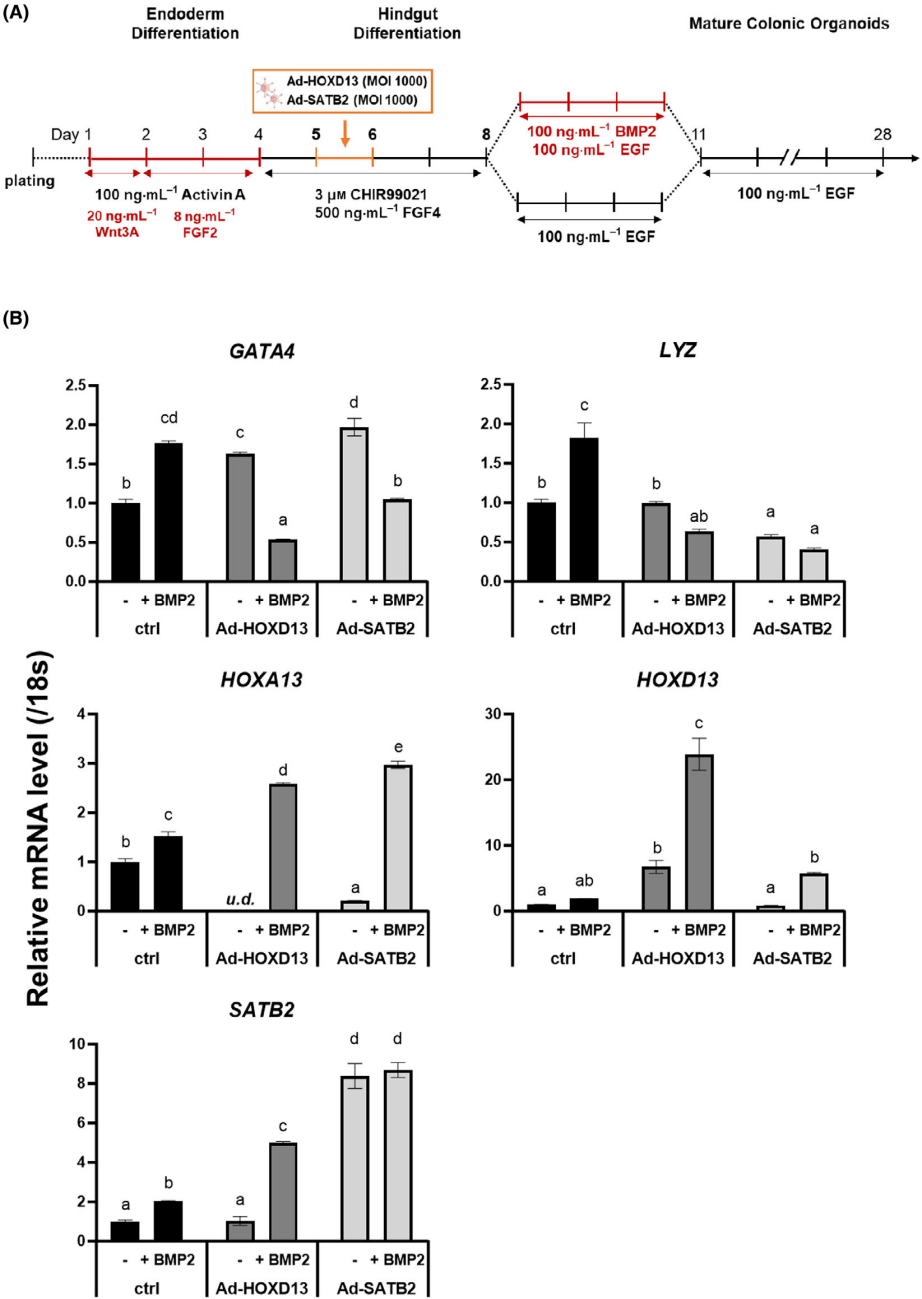
HOXD13 and SATB2 expression enhances BMP2‐mediated colonic fate specification during HCO development. (A) Schematic representation of differentiation protocol. (B) RT‐qPCR analysis of small intestine (*GATA4* and *LYZ*) and colon (*HOXA13*, *HOXD13*, and *SATB2*) markers on day 28 HCOs. TkDN4‐M‐derived differentiated cells were transduced with Ad‐HOXD13 or Ad‐SATB2 (MOI of 1000) on day 5. Spheroids were cultured with or without BMP2 for 3 days and subsequently maintained in colon‐differentiation medium until day 28. Experiments were performed independently twice (*n* = 2) with three biological replicates. Data were normalized to 18S rRNA and are presented as mean ± SEM. Statistical analysis was performed using the Tukey's test (distinct letters, *P* < 0.05). ‘*u.d*.’ indicates ‘undetectable’.

To elucidate whether transient expression of either HOXD13 or SATB2 during HCO differentiation could obviate the requirement for BMP2 treatment, which impedes organoid growth, the hindgut endoderm was transduced with Ad‐HOXD13 or Ad‐SATB2 at an MOI of 1000, followed by culture in the presence or absence of BMP2 during the three‐day patterning phase (Fig. 4A). RT‐qPCR analysis demonstrated that while transduction of HOXD13 or SATB2 alone was insufficient to replicate BMP signaling activation, it synergistically enhanced BMP2‐mediated promotion of colonic identity, which was revealed by the downregulation of small intestine markers (*GATA4* and *LYZ*) and upregulation of colon markers (*HOXA13*, *HOXD13*, and *SATB2*) (Fig. 4B). Collectively, our findings demonstrate that BMP2 signaling plays an essential role in establishing colonic identity, while the expression of HOXA13 and SATB2 during hindgut differentiation further enhances posterior gut fate specification.

## Discussion

In this study, we confirmed that BMP2 signaling is crucial for inducing colon signatures in developing hindgut spheroids, highlighting the superior potential of the CSC protocol for HCO differentiation. We further enhanced this process by transducing the progenitor cells with Ad‐HOXD13 or Ad‐SATB2, which augmented BMP2‐mediated colonic fate specification and resulted in increased maturity and colonic identity. These findings collectively showcase our development of an improved method to generate HCOs, potentially advancing their use in various research fields.

The development of the gastrointestinal tract depends on precise spatial regulation. One critical temporal instruction for patterning intestinal epithelium is conveyed through mesoderm‐endoderm interactions, which are mediated by several key signaling pathways at specific developmental stages [6, 8, 9]. Although long‐term *in vitro* cultures of primary adult human intestinal epithelial tissues require BMP signaling inhibition [30], our results corroborate previous findings that transient BMP2 signaling activation is crucial for conferring colonic identity to hindgut spheroids by inducing colon‐specific signatures [10]. Activation of BMP2 signaling is essential for primitive gut development and promotes enteric neuron maturation during large intestine development during embryogenesis [6, 31]. Importantly, our findings demonstrate that merely activating regional specification genes, namely, *HOXD13* and *SATB2*, is insufficient to promote colonic properties; rather, the presence of both spatial and temporal instructions enhances colonic fate determination. Additionally, given that the CSC protocol reportedly induces concurrent mesenchymal differentiation [10], further investigation would be needed to determine whether the enhanced colonic properties we observed are directly attributable to epithelial differentiation or potentially influenced by mesenchymal‐epithelial interactions during development. This consideration is particularly relevant, given the known role of mesenchymal‐epithelial crosstalk in intestinal development and patterning.

HOXD13 plays an indispensable role in the spatial regulation of colonic epithelial development. During embryogenesis, HOX genes exhibit a posterior prevalence phenomenon, wherein posterior HOX genes repress anterior HOX genes, ensuring proper spatial and temporal regulation [13]. This regulation is achieved through complex auto‐ and cross‐regulatory mechanisms within the HOX cluster [32], involving both positive and negative influences [33], which underscores the critical role of posterior HOX genes, such as HOXD13. In contrast, Hoxd13 has been implicated in several critical processes during limb patterning and skeletogenesis, including the initiation of chondrogenic condensation and interdigital cell death *via* BMP signaling [26]. Moreover, BMP2 has been identified as a direct target of Hoxd13 protein [34, 35]. Such a relationship between HOXD13 and BMP signaling in other developmental contexts provides insights into a potential mechanism for their synergistic effects on colonic fate patterning. While the specific interaction between SATB2 and BMP signaling is not well characterized, it potentially complements the spatial patterning effects of HOXD13, that is, these factors may cooperatively contribute to the complex network of spatial and temporal signals that guide colonic epithelial development and maintenance.

Although the HCOs generated in this study demonstrated elevated colonic identity in their gene profiles, several limitations should be addressed in future studies. First, we established and validated our refined differentiation protocol using an only single iPSC line (TkDN4‐M). To ensure the robustness and reproducibility of our findings, this protocol should be tested across multiple iPSC lines from different genetic backgrounds. Furthermore, since the efficiency of adenovirus‐mediated gene transduction in this experiment may be affected by slight differences in the efficiency of differentiation and the timing of spheroid appearance, and the extent of cytotoxicity may vary depending on the quality of the virus, one of the challenges in generalizing this method is the standardization so that inter‐experimental or individual differences are minimized. Additionally, further functional tests are required to confirm their physiological relevance. For instance, HCOs can be co‐cultured with pathogenic or protective microbes to simulate the complex microenvironment of the human colon. Examination of water and mineral absorption would ensure the mature function of the colon epithelium of HCOs. Regarding intestinal barrier functions, exposure of HCOs to stimuli such as short‐chain fatty acids can provide insights into their capacity to modulate mucin production and tight junction formation. These functional assays would validate the colonic identity of organoids and demonstrate their potential as robust models for studying colon biology and pathology.

In conclusion, our study showed that transient induction of HOXD13 or SATB2 during hindgut endoderm differentiation effectively enhanced its properties as colonic epithelium. Our findings would contribute to elucidating the key molecular determinants of colonic fate specification and provide a refined protocol for generating functionally mature human colon organoids from iPSCs.

## Author contributions

I‐TL: Assembly of data, data analysis, manuscript writing; YT: Conception and design, financial support, data interpretation, manuscript writing; TS, YY: Helpful discussion; RS: Financial support, final approval of manuscript.

### Peer review

The peer review history for this article is available at https://www.webofscience.com/api/gateway/wos/peer‐review/10.1002/1873‐3468.15082.

## Supporting information


**Fig. S1.** Immunofluorescence staining of GATA4 in iPSC‐derived organoids.
**Fig. S2.** Early stage Wnt3a and FGF2 supplementation enhanced spheroid production.
**Fig. S3.** Adenovirus production and temporal optimization of transduction during HCO differentiation.
**Table S1.** Differentiation medium used in the study.
**Table S2.** Probes and primers for RT‐qPCR.

## Data Availability

The datasets used in this study are available from the corresponding authors upon reasonable request.
