# Supplementary material for: Human colon organoid differentiation from induced pluripotent stem cells using an improved method
Source: FEBS Lett. 2024 Dec 23;599(6):912–24. doi: 10.1002/1873-3468.15082 (PMC11931984; doi:10.1002/1873-3468.15082)
Supplement: Supplementary file 1 — Fig. S1. Immunofluorescence staining of GATA4 in iPSC‐derived organoids. Fig. S2. Early stage Wnt3a and FGF2 supplementation enhanced spheroid production. Fig. S3. Adenovirus production and temporal optimization of transduction during HCO differentiation. Table S1. Differentiation medium used in the study. Table S2. Probes and primers for RT‐qPCR. [file FEB2-599-912-s001.pdf]

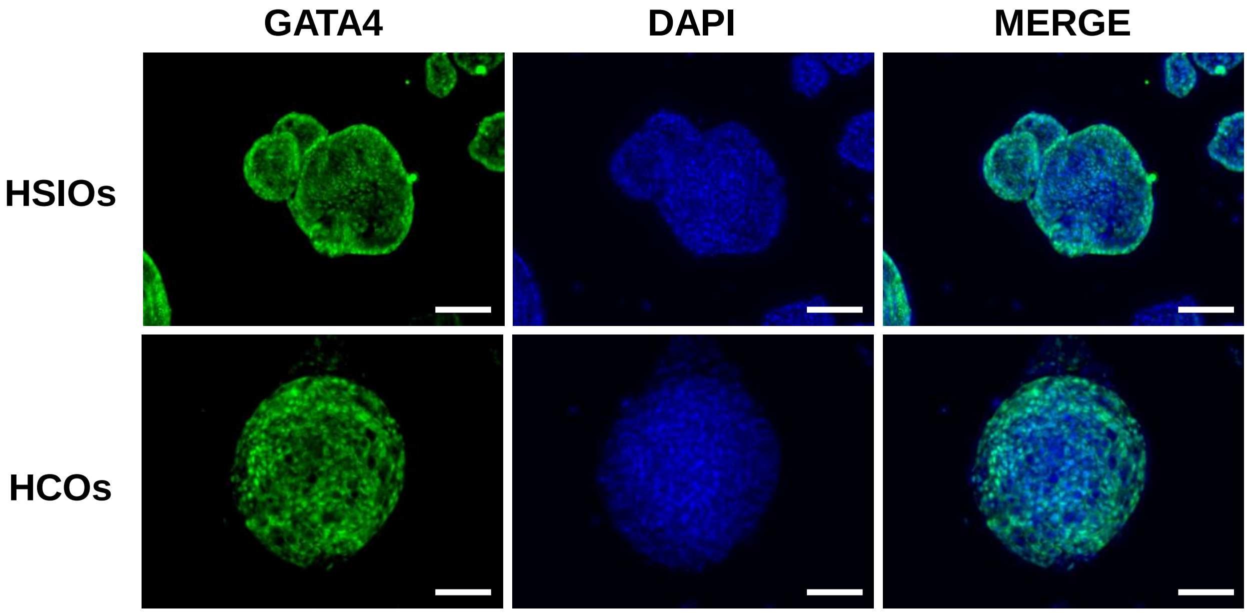

Fig. S1. Immunofluorescence staining of GATA4 in iPSC-derived organoids.
Comparative analysis of GATA4 expression in HSIOs and HCOs generated using the CSC protocol. Scale bar: 20 µm.

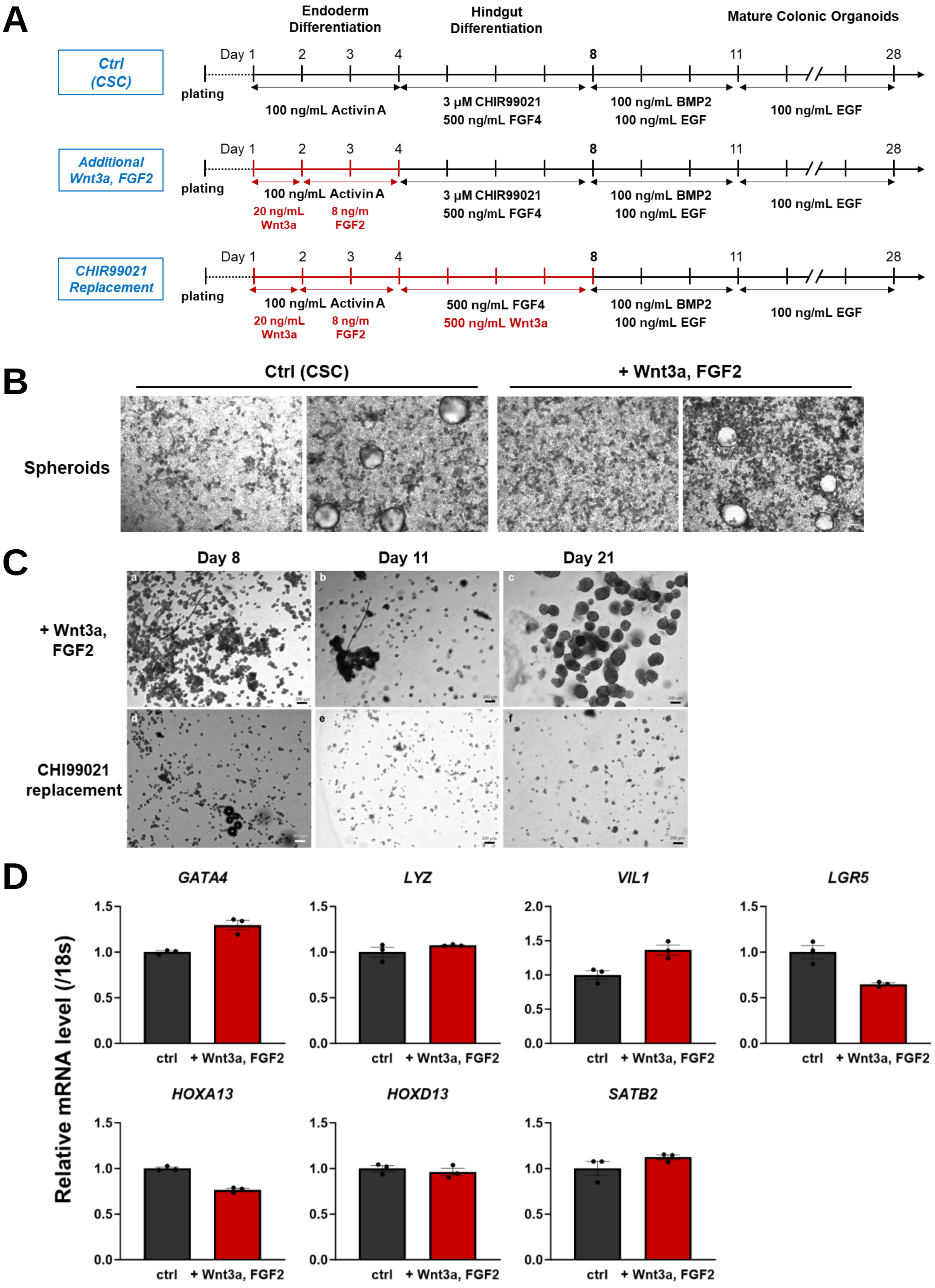

Fig. S2. Early stage Wnt3a and FGF2 supplementation enhanced spheroid production. (A) Schematic representation of the differentiation protocol, showing Wnt3a and FGF2 supplementation during definitive endodermal induction (days 1–3) and the replacement of CHIR99021 with Wnt3a during hindgut patterning (days 4–8). (B) Phase-contrast images on day 8 after differentiation of TkDN4-M, showing attached monolayers and floating spheroids (visible as black dots). Scale bar = 200 μm. (C) Bright-field images of differentiating HCOs derived from the modified protocols in (A) on days 8, 11, and 21. Scale bar = 200 μm. (D) RT-qPCR analysis of small intestine (*GATA4* and *LYZ*), intestinal epithelium (*VIL1*), intestinal stem cells (*LGR5*), and colon (*HOXA13*, *HOXD13*, *SATB2, and MUC2*) markers on day 28 HCOs. Experiments were performed independently three times (n = 3) with three biological replicates. Data normalized to 18s rRNA are shown as the mean ± SEM. The closed black circles in the graph indicate individual data points.

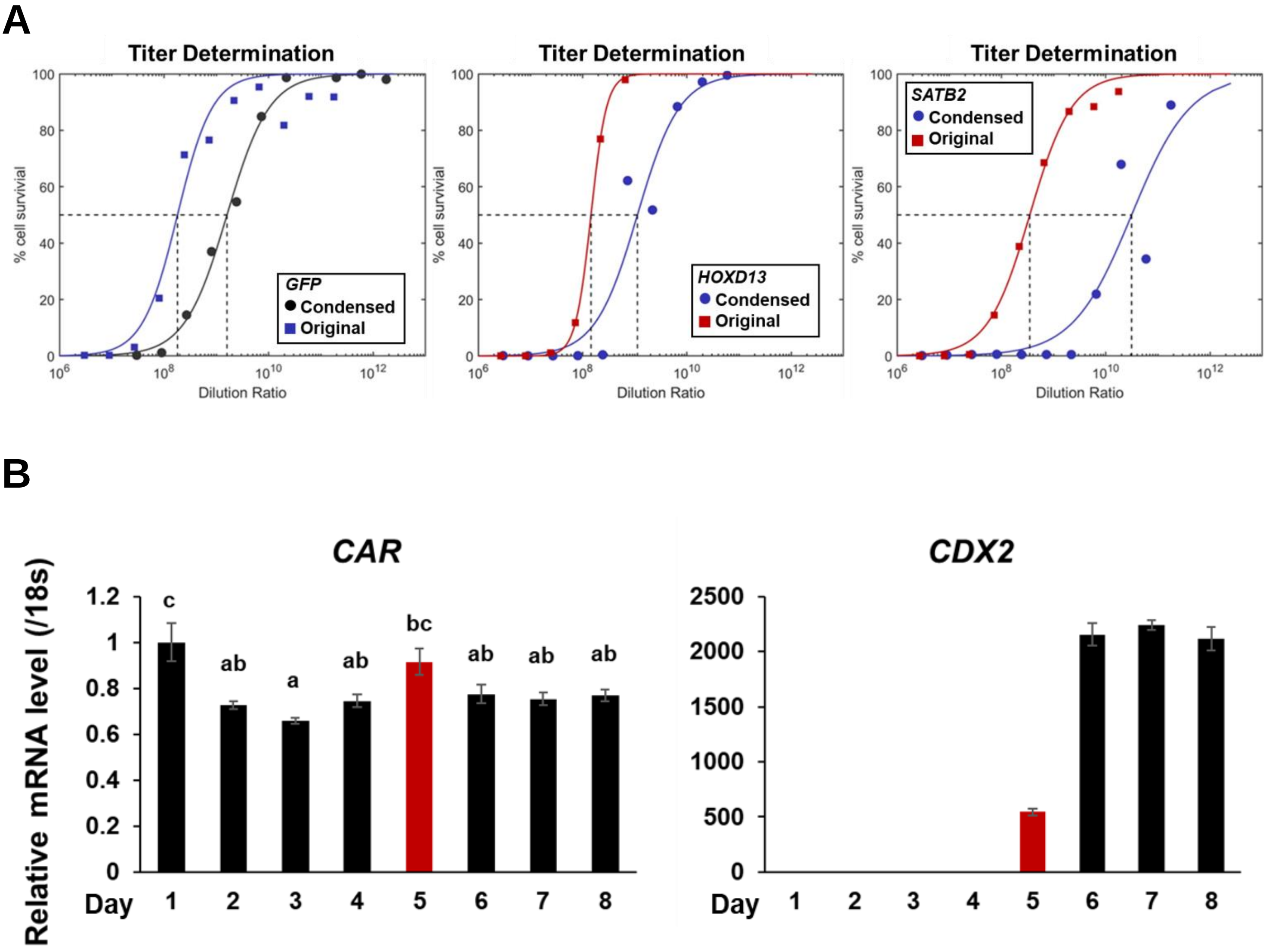

Fig. S3. Adenovirus production and temporal optimization of transduction during HCO differentiation. (A) Adenoviral stock purification using cesium chloride gradient ultracentrifugation and titer determination by a cell viability assay using CellTiter-Glo reagents. (B) RT-qPCR analysis of adenoviral receptor *CXADR* and hindgut endoderm marker *CDX2* during TkDN4-M differentiation following the modified Cell Stem Cell protocol. Experiments were performed independently three times ($n = 3$) with three biological replicates. Data normalized to 18s rRNA are shown as the mean ± SEM. Statistical analysis was performed using the Tukey's test (distinct letters, $p < 0.05$).

Table S1. Differentiation medium used in the study.

Protocol	Induction stage	Recipe
HCOs<br>(CSC)	Definitive endoderm	Day 1: 100 ng/mL Activin A in BM1<br>Day 2: 100 ng/mL Activin A, 0.2% defined FBS in BM1<br>Day 3: 100 ng/mL Activin A, 2% defined FBS in BM1
	Hindgut	Day 4–7: 3 μM CHIR99021, 500 ng/mL hFGF4, 2% defined FBS in BM1<br>Medium was refreshed daily.
	Colon	Day 8–10: 100 ng/mL BMP2, 100 ng/mL mEGF, 1x B27, 1x N2, 15 mM HEPES in BM2<br>Medium was refreshed daily.<br>Day 11–28: 100 ng/mL mEGF, 1x B27, 1x N2, 15 mM HEPES in BM2<br>Medium was refreshed twice a week.
HCOs<br>(NM)	Definitive endoderm	Day 1: 100 ng/mL Activin A, 3 μM CHIR99021 in BM1<br>Day 2–3: 100 ng/mL Activin A, 0.2% BSA in BM1<br>Medium was refreshed daily.
	Hindgut	Day 4–7: 3 μM CHIR99021, 500 ng/mL hFGF4, 1x B27 in BM1<br>Medium was refreshed daily.
	Colon	Day 8–48: 1x B27, 3 μM CHIR99021, 300 nM LDN 193189, 100 ng/mL mEGF in BM2<br>Medium was refreshed every 2 days.
HIOs<br>(Stem Cell Report)	Definitive endoderm	Day 1: 100 ng/mL Activin A, 20 ng/mL hWnt3A in BM1<br>Day 2: 100 ng/mL Activin A, 8 ng/mL hFGF2, 0.2% defined FBS in BM1<br>Day 3: 100 ng/mL Activin A, 8 ng/mL hFGF2, 2% defined FBS in BM1
	Hindgut	Day 4–7: 500 ng/mL hWnt3A, 500 ng/mL hFGF4, 2% defined FBS in BM1<br>Medium was refreshed daily.
	Small intestine	Day 8–21: 100 ng/mL Noggin, 100 ng/mL mEGF, 1x B27, 1x N2, 15 mM HEPES, 500 ng/mL mR-Spondin1 in BM2
HCOs<br>(Modified-CSC)	Definitive endoderm	Day 1: 100 ng/mL Activin A, 20 ng/mL hWnt3A in BM1<br>Day 2: 100 ng/mL Activin A, 8 ng/mL hFGF2, 0.2% defined FBS in BM1<br>Day 3: 100 ng/mL Activin A, 8 ng/mL hFGF2, 2% defined FBS in BM1
	Hindgut	Day 4–7: 3 μM CHIR99021, 500 ng/mL hFGF4, 2% defined FBS in BM1<br>Medium was refreshed daily.
	Colon	Day 8–10: 100 ng/mL BMP2, 100 ng/mL mEGF, 1x B27, 1x N2, 15 mM HEPES in BM2<br>Medium was refreshed daily.<br>Day 11–28: 100 ng/mL mEGF, 1x B27, 1x N2, 15 mM HEPES in BM2<br>Medium was refreshed twice a week.

Basal Medium 1 (BM1) for definitive endoderm and hindgut differentiation was prepared by supplementing RPMI-1640 (Gibco) with 1% (v/v) Penicillin-Streptomycin-Glutamine (100X, Gibco).

Basal Medium 2 (BM2) for intestinal organoid differentiation was prepared by supplementing Advanced DMEM/F-12 (Gibco) with 1% (v/v) Penicillin-Streptomycin-Glutamine (100X, Gibco).

Table S2. Probes and primers for RT-qPCR.

Probe		
Gene	Supplier	ID
GATA4	IDT	Hs.PT.58.259457
LYZ	IDT	Hs.PT.58.24761205
VIL1	IDT	Hs.PT.58.4630053
LGR5	IDT	Hs.PT.58.227062
HOXA13	IDT	Hs.PT.58.22565110
HOXD13	IDT	Hs.PT.58.3026351
SATB2	IDT	Hs.PT.58.24560574
CXADR	IDT	Hs.PT.58.904381.g
18s	IDT	Hs.PT.39a.22214856.g

Primer		
Gene	Forward	Reverse
CDX2	5'-ACTACAGTCGCTACATCACCA-3'	5'-TTTTCCTCTCCTTTGCTCTGC-3'
gfp	5'-CACATGAAGCAGCACGACTT-3'	5'-TTGCCATCCTCCTTGAAATC-3'
